# Soluble CD36 Ectodomain Binds Negatively Charged Diacylglycerol Ligands and Acts as a Co-Receptor for TLR2

**DOI:** 10.1371/journal.pone.0007411

**Published:** 2009-10-22

**Authors:** Maximiliano J. Jimenez-Dalmaroni, Nengming Xiao, Adam L. Corper, Petra Verdino, Gary D. Ainge, Dave S. Larsen, Gavin F. Painter, Pauline M. Rudd, Raymond A. Dwek, Kasper Hoebe, Bruce Beutler, Ian A. Wilson

**Affiliations:** 1 Glycobiology Institute, Department of Biochemistry, University of Oxford, Oxford, United Kingdom; 2 Department of Molecular Biology and the Skaggs Institute for Chemical Biology, The Scripps Research Institute, La Jolla, California, United States of America; 3 Department of Genetics, The Scripps Research Institute, La Jolla, California, United States of America; 4 Industrial Research Limited, Carbohydrate Chemistry, Lower Hutt, New Zealand; 5 University of Otago, Dunedin, New Zealand; 6 Division of Molecular Immunology, Cincinnati Children's Hospital Research Foundation, Cincinnati, Ohio, United States of America; New York University School of Medicine, United States of America

## Abstract

**Background:**

Cluster of differentiation 36 (CD36) is a transmembrane glycoprotein involved in many biological processes, such as platelet biology, angiogenesis and in the aetiopathology of atherosclerosis and cardiovascular diseases. Toll-like receptors (TLRs) are one of the most important receptors of the innate immune system. Their main function is the recognition of conserved structure of microorganisms. This recognition triggers signaling pathways that activate transcription of cytokines and co-stimulatory molecules which participate in the generation of an immune response against microbes. In particular, TLR2 has been shown to recognize a broad range of ligands. Recently, we showed that CD36 serves as a co-receptor for TLR2 and enhances recognition of specific diacylglycerides derived from bacteria.

**Methodology/ Principal Findings:**

Here, we investigate the mechanism by which CD36 contributes to ligand recognition and activation of TLR2 signaling pathway. We show that the ectodomain of murine CD36 (mCD36ED) directly interacts with negatively charged diacylglycerol ligands, which explains the specificity and selectivity of CD36 as a TLR2 co-receptor. We also show that mCD36ED amplifies the pro-inflammatory response to lipoteichoic acid in macrophages of wild-type mice and restores the pro-inflammatory response of macrophages from mice deficient in CD36 (*oblivious*), but not from mice deficient in cluster of differentiation 14 (CD14) (*heedless*).

**Conclusion/ Significance:**

These data indicate that the CD36 ectodomain is the only relevant domain for activation of TLR2 signaling pathway and that CD36 and CD14 have a non-redundant role for loading ligands onto TLR2 in the plasma-membrane. The pro-inflammatory role of soluble CD36 can be relevant in the activation of the immune response against pathogens, as well as in the progression of chronic diseases. Therefore, an increased level of soluble forms of CD36, which has been reported to be increased in type II diabetic patients, could accelerate atherosclerosis by increasing the pro-inflammatory response to diacylglycerol ligands.

## Introduction

CD36, the prototype of scavenger receptor class B, is a multifunctional transmembrane glycoprotein, involved in the aetiopathology of several biological processes and diseases, including cardiovascular diseases, Alzheimer's disease, *Plasmodium falciparum* (*P. falciparum*) infection, diabetes, angiogenesis, platelet biology, atherosclerosis [1]–[3], and anti-tumor responses [4]. The role of CD36 in these processes is attributed to its ability to bind a broad range of ligands [5]. Among these are long-chain fatty acids [6], advanced glycosylation products [7], [8], oxidized Low Density Lipoproteins [9]–[11], oxidized phosphocholines [12], collagen [13], growth-hormone releasing hormone (GHRH) peptides hexarelin and EP80317 [14], and thrombospondin-1 (TSP-1) [15].

Recently, we demonstrated that mice carrying a missense mutation in CD36, termed *oblivious*, had a reduced response to specific TLR2 ligands, lipoteichoic acids (LTA) and macrophage activating lipoprotein-2 (MALP-2), but responded normally to the synthetic lipopeptides, Pam_3_CSK_4_ and Pam_2_CSK_4_ [16]. Moreover, CD36*^obl/obl^* mice were unable to clear *Staphylococcus aureus* (*S. aureus*) bacteria and showed higher mortality compared to C57BL/6 mice. *Cd36−/−* mice were also more prone to develop abscesses [17]. A recent report also revealed that CD36-deficient mice showed impaired tumor necrosis factor-alpha (TNF-α) secretion in response to glycosylphosphatidylinositol (GPI) from *P. falciparum* and higher parasitemia levels and mortality when infected with *Plasmodium chabaudi chabaudi* AS (*P. chabaudi chabaudi* AS) [18]. Although these data highlight the role of CD36 in controlling infection with *S. aureus* and *P. falciparum*, the mechanism by which CD36 contributes to TLR2 signaling pathway activation has been elusive. Recent reports have attempted to address this issue, but with contrasting findings. One study [17] initially demonstrated requirement of the CD36 C-terminus for endocytosis and nuclear factor kappa-light-chain-enhancer of activated B cells (NF-κB) activation, whereas other reports showed that internalization of LTA is not necessary for signal transduction [19], [20].

Here, we address the role of CD36 in the TLR2-dependent activation of the myeloid differentiation protein 88 (MyD88) signaling pathway. To investigate the role of CD36 as a TLR2 co-receptor, the ectodomain of murine CD36 (mCD36ED) was cloned and expressed using a baculovirus expression system. The interaction of mCD36ED with TLR2 ligands and the effect of mCD36ED on the pro-inflammatory response to LTA by primary macrophages isolated from mice with non functional CD36 (*oblivious*) [16], CD14 (*heedless*) [21], TLR6 (*insouciant*) [22], knock-out mice (*Tlr2−/−, Tlr1−/−*), and wild-type (C57BL/6) mice were determined.

## Results

### mCD36ED expresses in insect cells as a monomer with α-helical and β-sheet secondary structure

**Figure 1:**
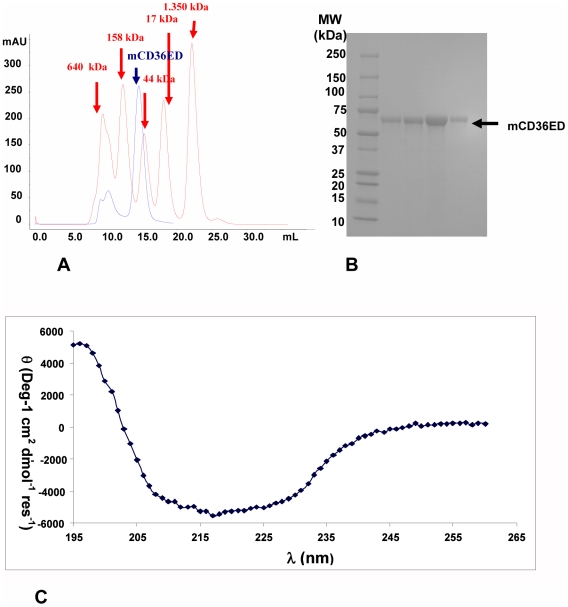
Expression, purification, and characterization of mCD36ED. (A) mCD36ED is an apparent monomer in size exclusion chromatography. mCD36ED and molecular weight standards (depicted in red) were run on a Superdex 200 10/30 gel filtration column. mCD36ED elutes at 14.7 ml which corresponds to the retention volume for a protein of ∼52 kDa and roughly corresponds to a molecular weight of 58.3 kDa of mCD36ED as determined by MALDI TOF MS. (B) Fractions of mCD36ED after size exclusion chromatography were run on an SDS PAGE. The position of purified mCD36ED, which runs with an apparent molecular weight between 50 and 75 kDa, is indicated. (C) mCD36ED is folded. The CD spectrum of mCD36ED was recorded at 25°C. Triplicate measurements were averaged, the CD spectrum of the buffer subtracted, and the CD data were converted from millidegrees to molar ellipticity per residue. Typical CD spectra of proteins rich in α-helical, β-sheet, and mixed α/β secondary structure are shown for comparison. α-helical proteins typically display a CD spectrum dominated by a strong maximum around 190 nm and two minima at 208 nm and 222 nm, while β-sheet proteins show a less pronounced maximum at 195 nm and a minimum around 215 nm. The CD spectrum of mCD36ED with its broad minimum between 207-230 nm and a maximum around 195 nm is thus indicative of the presence of both α-helices and β-sheets in its secondary structure.

mCD36ED DNA, amplified by PCR with a hexa-histidine tag at its carboxyl-terminus (C-terminal His-tag), was cloned in a baculovirus transfer vector and mCD36ED protein was expressed using a baculovirus/insect cell expression system. Protein expression was confirmed with an anti-His-tag Western blot and peptide identification using mass spectrometry (Supplementary Figure S1). mCD36ED ran as a monomer on size exclusion chromatography (Figures 1A, 1B) and its CD spectrum at 25°C revealed a broad minimum from 207 to 230 nm and a maximum at 195 nm, indicative of a folded protein containing α-helices and β-sheets in its secondary structure [23]. Typical CD spectra of proteins rich in α-helical, β-sheet, and mixed α/β secondary structure are also shown for comparison (Figure 1C).

### mCD36ED has intramolecular disulfide bonds and N-linked glycans

Under reducing condition, the electrophoretic mobility of mCD36ED is altered which indicates the presence of intramolecular disulfide bonds (Supplementary Figure S2A). N-linked glycosylation can be partially removed from mCD36ED by treatment with Peptide-N-glycosidase F (PNGase F) in native conditions to the same extent as in denaturing conditions, revealing that its N-glycosidic linkages are accessible for enzymatic cleavage (Supplementary Figure S2B). However, PNGase F was not able to release all glycans from mCD36ED (i.e., the theoretical molecular weight of mCD36ED is 47,424 kDa, but after digestion with PNGase F, mCD36ED runs at a molecular weight above 50 kDa). mCD36ED were expressed in Hi-5 insect cells, which have been reported to modify recombinant glycoproteins by adding a core (α 1→3) fucose [24], [25]. Therefore, the resistance to PNGase F treatment could be explained by the presence of core fucosylation in N-linked glycans of mCD36ED. The position of disulfide bonds based on bovine CD3*6* [26] and predicted N-linked glycosylation sites are depicted in Supplementary Figure S2C.

### mCD36ED binds to LTA, FSL-1, Pim2, and Pim4

**Figure 2:**
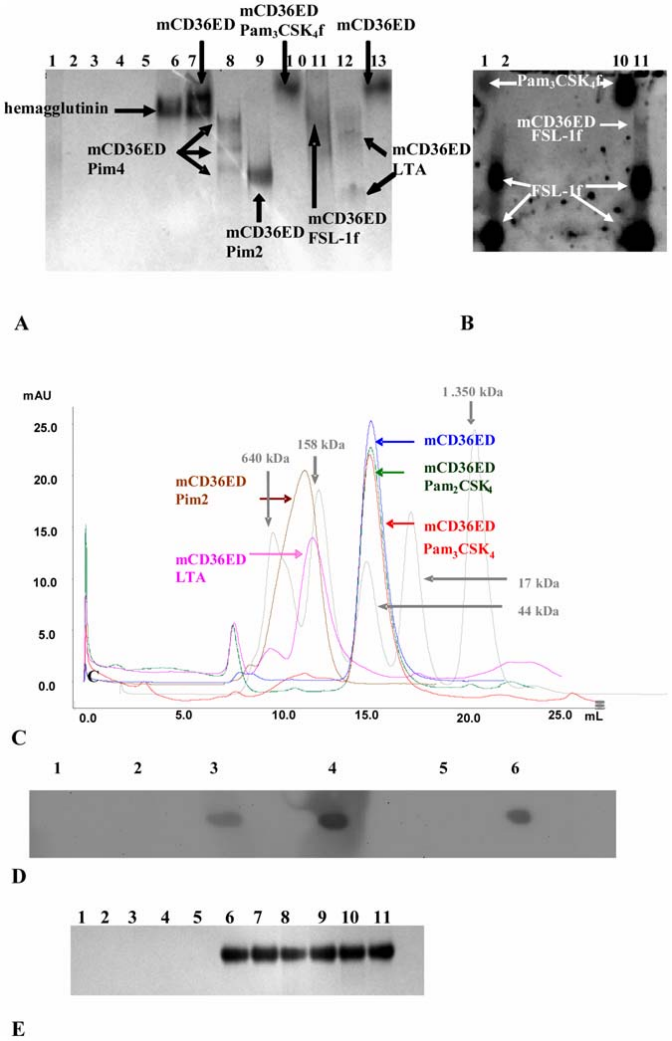
mCD36ED binds to FSL-1, Pim2, LTA and Pim4, but not to Pam_3_CSK_4_. mCD36ED was incubated overnight with TLR2 ligands or influenza hemagglutinin (H5N1 Viet 04 strain) and subsequently run on a native 4–20% PAGE for 4 hours at 100 volts. Fluorescence was determined with a Versadoc imaging system. Subsequently, the gel was stained with Coomassie Blue. (A) Native gel stained with Coomassie Blue. (B) Fluorescence from the native gel determined using a Versadoc imaging system. Lanes: 1- 1 µg of Pam_3_CSK_4_- fluorescein (Pam_3_CSK_4_f) ; 2- 1 µg of FSL-1-fluorescein (FSL-1f); 3- 1 µg of Pim4; 4- 1 µg of Pim2; 5- 1 µg of LTA; 6- 10 µg of hemagglutinin Viet 04; 7- 10 µg of mCD36ED incubated with 10 µg of hemagglutinin Viet 04; 8- 10 µg of mCD36ED incubated with 1 µg of Pim4; 9- 10 µg of mCD36ED incubated with 1 µg of Pim2; 10- 10 µg of mCD36ED incubated with 1 µg of Pam_3_CSK_4_f; 11- 10 µg of mCD36ED incubated with 1 µg of FSL-1f 12- 10 µg of mCD36ED incubated with 1 µg of LTA; 13- 10 µg of mCD36ED. (C) The retention volume of mCD36ED-LTA and mCD36ED-Pim2 is decreased as compared to uncomplexed mCD36ED. An appreciable shift in molecular weight is seen after incubation of mCD36ED with LTA or Pim2, but no shift is observed with Pam_2_CSK_4_ or Pam_3_CSK_4_. Molecular weight standards were run in parallel to determine the apparent change in molecular weight of mCD36ED. (D) The presence of LTA in the mCD36ED-LTA size exclusion chromatography fractions was confirmed by dot blot analysis using an anti-LTA antibody. Lanes 1- PBS; 2- Pam_2_CSK_4_; 3- anti-LTA antibody; 4- LTA; 5- mCD36ED; 6- mCD36ED- LTA. (E) KRN TCR with a C-terminal His-tag does not bind ligands of TLR2. Lanes 1- 1 µg of Pam_3_CSK_4_; 2- 1 µg of LTA; 3- 1 µg of FSL-1, 4- 1 µg of Pim2, 5- 1 µg of Pim4, 6- 10 µg of KRN TCR; 7- 10 µg of KRN TCR incubated with 1 µg of Pam_3_CSK_4_; 8- 10 µg of KRN TCR incubated with 1 µg of LTA, 9- 10 µg of KRN TCR incubated with 1 µg of FSL-1, 10- 10 µg of KRN TCR incubated with 1 µg of Pim2, 11- 10 µg of KRN TCR incubated with 1 µg of Pim4.

We then demonstrated by native PAGE assays that mCD36ED binds to LTA from *S. aureus,* synthetic phosphatidyl-myo-inositol mannosides (Pims) with two (Pim2) and four mannoses (Pim4) from mycobacteria, synthetic fibroblast stimulating lipopeptide-1 with a fluorescein tag (FSL-1-fluorescein) from *Mycoplasma salivarium*, but not to Pam_3_CSK_4_ (Figures 2A, 2B). Binding of mCD36ED to FSL-1-fluorescein was also confirmed by fluorescence (Figure 2B). As a negative control, we employed influenza hemagglutinin from the H5N1 Viet 04 strain [27], which lacks diacylglycerol moieties and was unable to interact with human TLR2 ectodomain (data not shown). Moreover, although Pam_2_CSK_4_ and Pam_3_CSK_4_ did not alter the retention volume of mCD36ED, binding of both Pim2 and LTA produced a shift to higher molecular weights in size exclusion chromatography (Figure 2C). Dot blot experiments using an antibody specific to the polyglycerophosphate moiety of LTA confirmed its presence in the mCD36ED-LTA complex (Figure 2D). Furthermore, we employed a T-cell receptor (TCR) termed KRN [28], which has a C-terminal His-tag, to discard the possibility of an non-specific interaction between the C-terminal His-tag and TLR2 ligands. The lack of binding of Pam_3_CSK_4,_ FSL-1 and LTA by KRN TCR excluded the possibility that the C-terminal His-tag is involved in the binding of TLR2 ligands (Figure 2E).

### Lipomannan, FSL-1-fluorescein, and LTA activation of the MyD88 pathway is dependent on TLR2, CD36, and CD14

**Figure 3:**
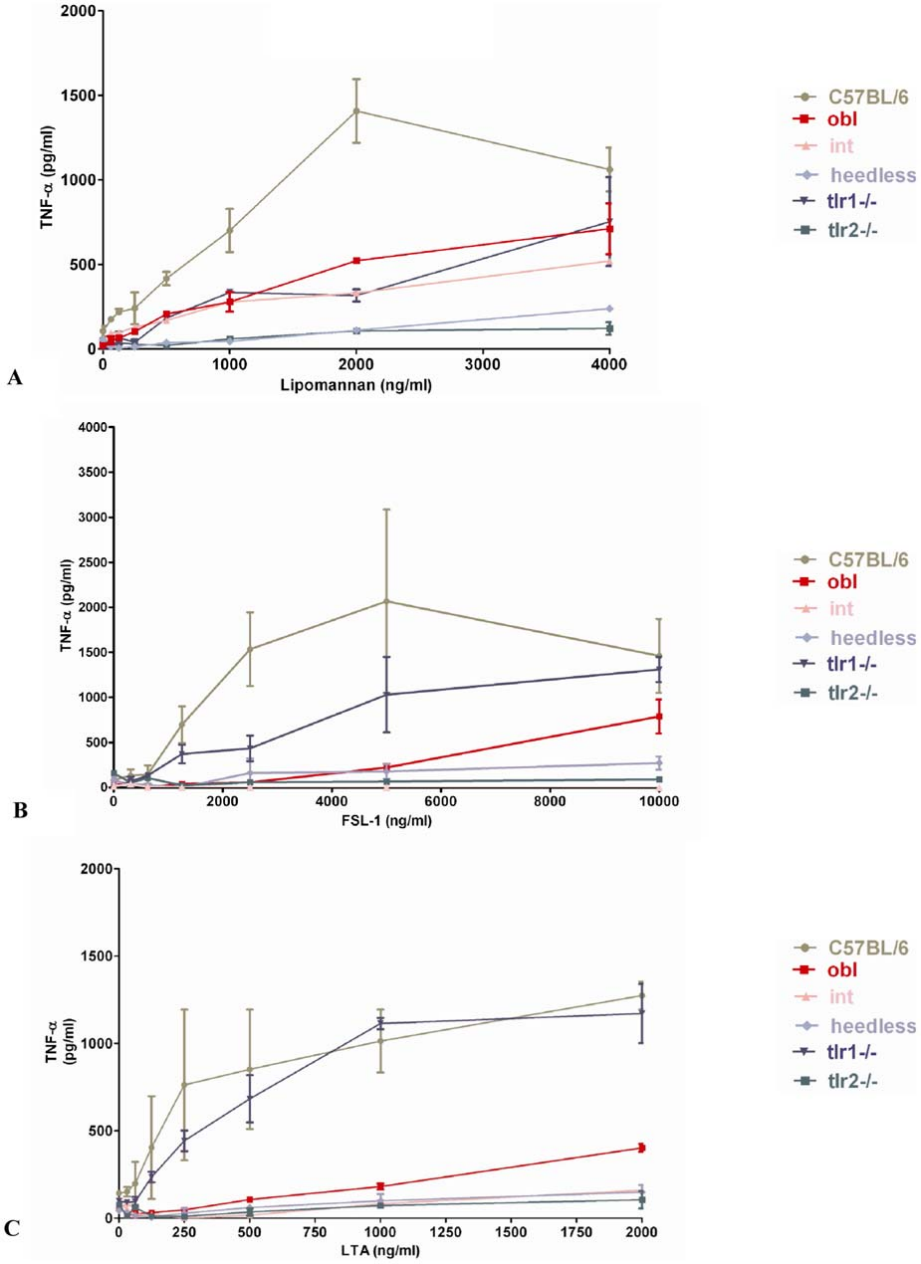
Expression of TLR2, CD14 and CD36 in murine macrophages are relevant for secretion of TNF-α by the following bacterial components: (A) lipomannan from *M. smegmatis,* (B) FSL-1 fluorescein, and (C). LTA from *S. aureus*. Macrophages from C57BL/6 mice, *heedless*, *insouciant* (int), *oblivious* (obl), *Tlr2−/−*, *Tlr1−/−* were exposed to different concentrations of lipomannan from *M. smegmatis,* LTA from *S. aureus* and FSL-1 fluorescein for 4 hours at 37°C. The amount of TNF-α secreted to the supernatant was determined by an L929 cell cytotoxic assay. Values are expressed as mean values +/− SEM (n = 2 mice).

To determine which receptors are important for the biological activity of lipomannan, FSL-1 and LTA, we employed wild-type C57BL/6, *insouciant* (TLR6*^int^*), *oblivious* (CD36*^obl^*), *heedless* (CD14*^hdl^*), *Tlr1* ^−/−^ and *Tlr2* ^−/−^ mice. *Insouciant, oblivious and heedless* were generated by germ-line, random mutagenesis using ENU (N-ethyl N-nitrosourea) where their phenotypes result from loss of functional TLR6, CD36 and CD14, respectively [22], [16], [21]. Macrophages isolated from *heedless, oblivious, insouciant* and *Tlr1*−/− mice were incubated with different concentrations of LTA for 4 hours. While macrophage secretion of TNF-α by FSL-1-fluorescein and LTA, were dependent on TLR6, macrophage activation by lipomannan from *M. smegmatis* was dependent on TLR6 and TLR1. Furthermore, TNF-α secretion by macrophages at low concentrations of the ligands was dependent on CD36, while the absence of TLR2 and CD14 impaired TNF-α secretion by macrophages even at higher ligand concentrations of LTA, FSL-1-fluorescein and lipomannan (Figure 3).

### mCD36ED amplifies the response to LTA in wild-type macrophages and restores the response to LTA in CD36-deficient, but not in CD14-deficient macrophages

**Figure 4:**
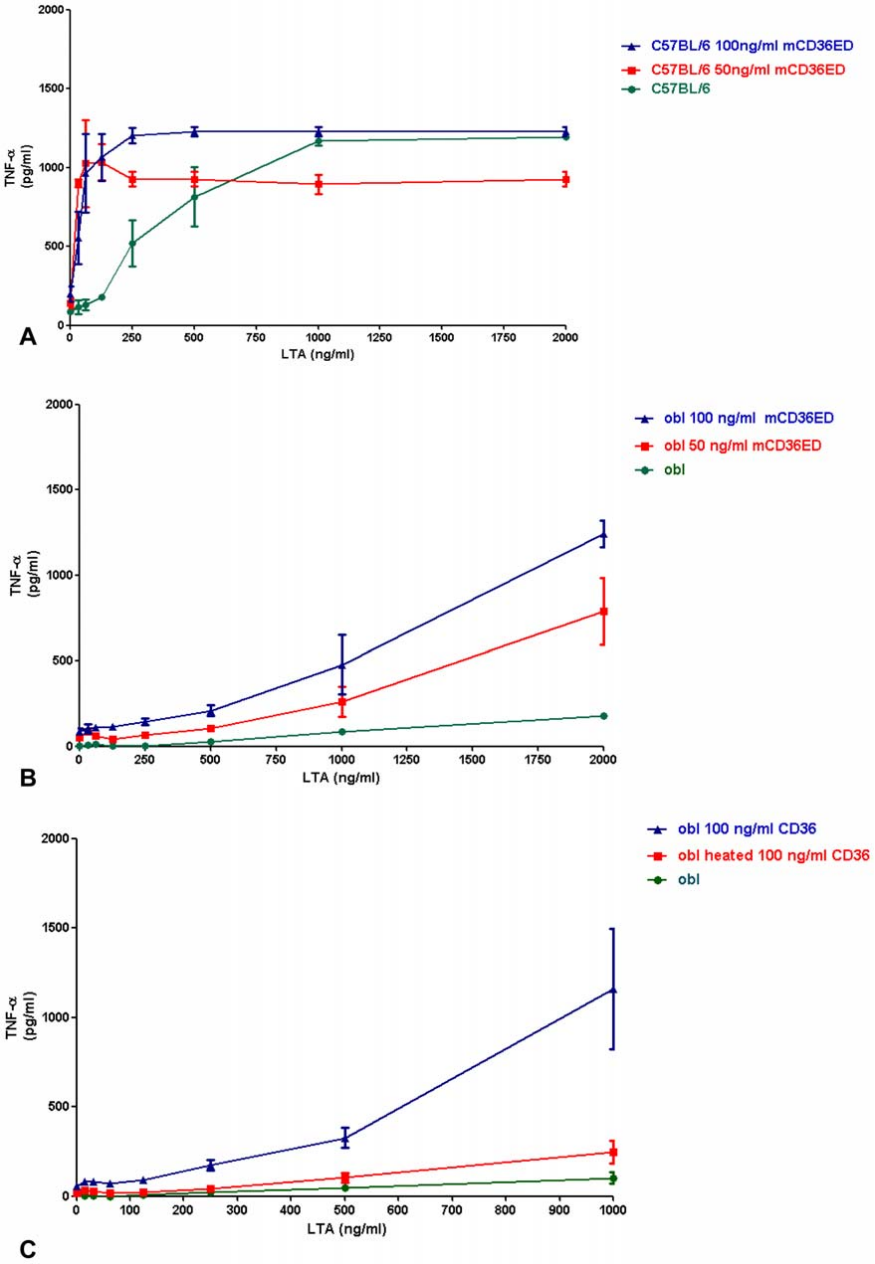
TNF-α secretion by macrophages with LTA. (A) mCD36ED increases secretion of TNF-α by low LTA concentrations in C57BL/6 macrophages. (B) mCD36ED restores secretion of TNF-α in *oblivious* (obl) macrophages. (C) Denatured mCD36ED did not restore secretion of TNF-α by *oblivious* (obl) macrophages. Macrophages from C57BL/6 and *oblivious* were exposed to different concentrations of LTA plus a constant amount of mCD36ED, or heated denatured mCD36ED (for *oblivious* macrophages) for 4 hours at 37°C. The amount of TNF-α secreted to the supernatant was determined by L929 cytotoxic assay. Values are expressed as mean values +/−_SEM (n = 3 mice).

**Figure 5:**
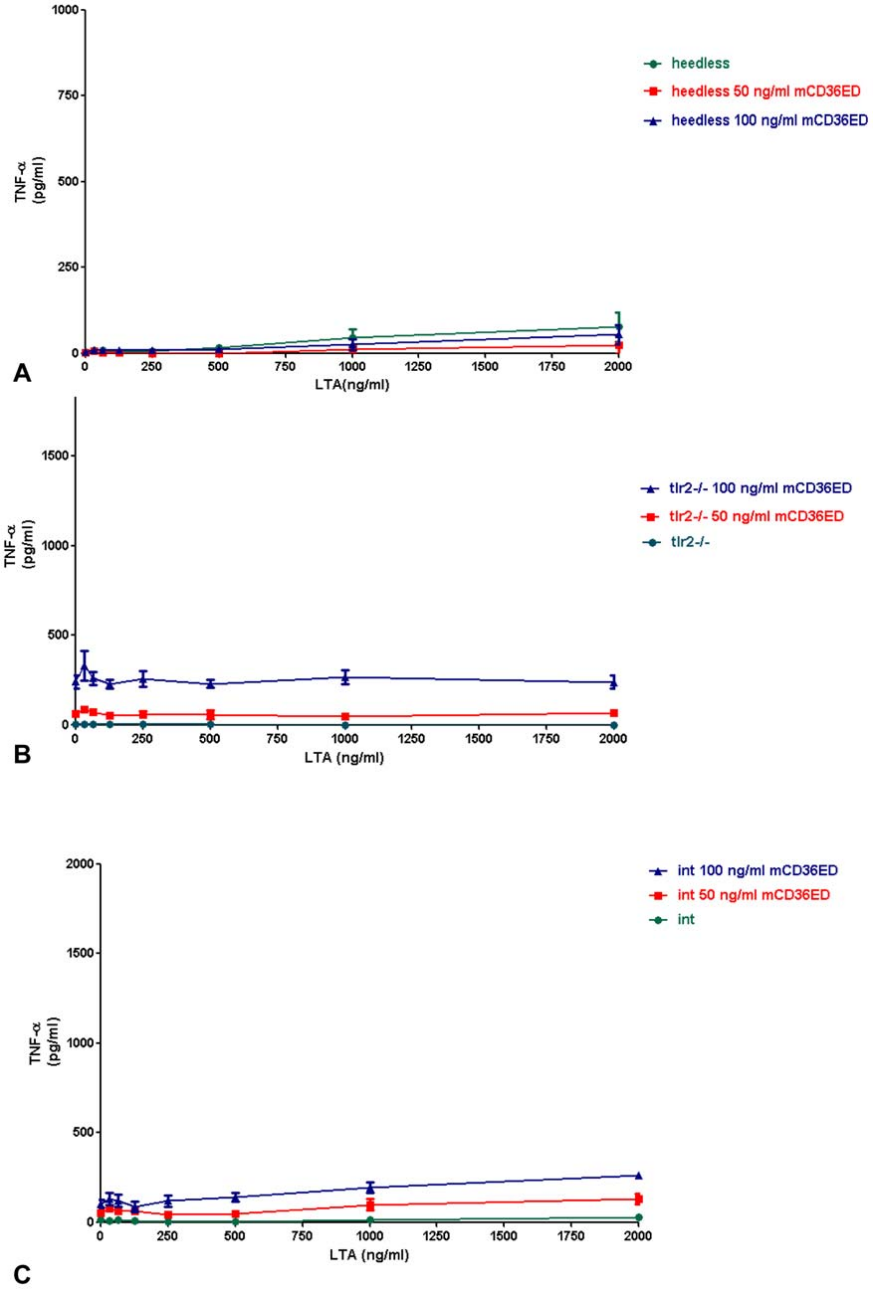
mCD36ED does not rescue deficient TNF-α secretion by LTA in the case of macrophages extracted from (A) *heedless*, (B) *tlr2−/−* and (C) *insouciant* (int) mice. Macrophages from *tlr2−/−, heedless* and *insouciant* macrophages were exposed to different concentration of LTA plus a constant amount of mCD36ED for 4 hours. The amount of TNF-α secreted to the supernatant was determined by an L929 cytotoxic assay. Values are expressed as mean values +/− SEM (n = 3 mice).

To further explore the role of CD36 in activation by TLR2 ligands, we examined the effect of mCD36ED on increasing amounts of LTA *in vitro* by measuring TNF-α production in primary cultured peritoneal macrophages. Addition of mCD36ED (50 and 100 ng/ml) increased macrophage sensitivity in C57BL/6 mice at low concentrations of LTA and restored the LTA activity to wild-type levels in CD36*^obl/obl^* mice (Figures 4A, 4B). Moreover, denaturation of mCD36ED, by heating for 2 hours at 100°C, abolished restoration of the response of *oblivious* mice to LTA (Figure 4C). In contrast, addition of mCD36ED only slightly increased TNF-α secretion by *insouciant* macrophages, and no significant increase of TNF-α secretion was observed for *Tlr2* ^/−^ and *heedless* macrophages (Figure 5).

### mCD36ED does not interact directly with mTLR2ED

**Figure 6:**
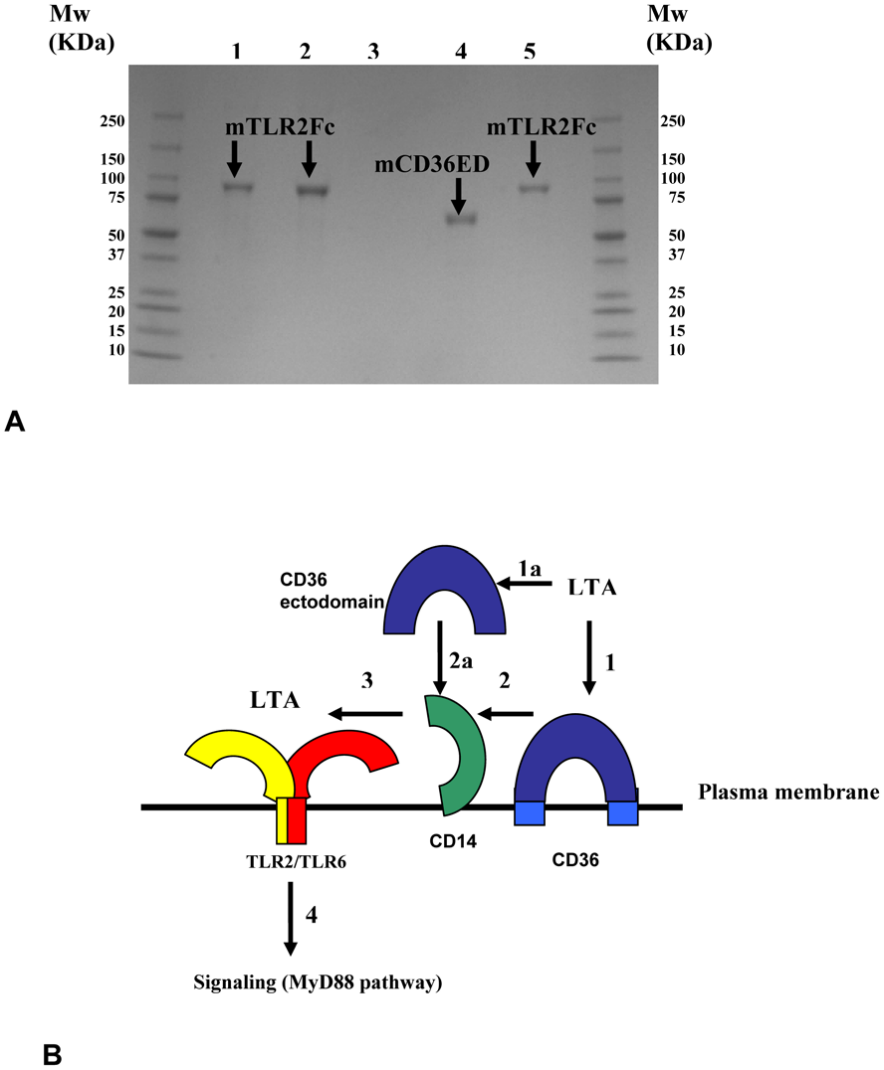
mCD36ED and TLR2. (A) mCD36ED does not interact with an mTLR2ED/Fc chimera. SDS PAGE of the samples from immunoprecipitation using protein G beads. Lanes: 1- Protein G incubated with mTLR2ED/Fc chimera and mCD36ED; 2- Protein G incubated with mTLR2ED/Fc chimera alone; 3- Protein G incubated with mCD36ED; 4- mCD36ED; 5- mTLR2ED. (B) Model of CD36-dependent activation of the TLR2 signaling pathway. CD36 binds LTA (step 1) which is transferred to CD14 (step 2). Alternatively, the soluble ectodomain of CD36 binds LTA (step 1a), and transfers it to CD14 (step 2a). Subsequently, CD14 transfers LTA to TLR2/TLR6 (step 3) and the MyD88 pathway is activated (step 4).

We investigated by immunoprecipitation whether direct interaction of mCD36ED occurs with a murine TLR2 ectodomain fused with an Fc domain of human IgG1 (mTLR2ED/Fc). We incubated mCD36ED with mTLR2ED/Fc and subsequently with magnetic protein G beads. After washing, samples from the immunoprecipitation were run on an SDS-PAGE. No immunoprecipitation of mCD36ED with mTLR2ED/Fc was observed (Figure 6A), suggesting that mCD36ED does not interact directly with mTLR2ED.

## Discussion

CD36 is a type III transmembrane glycoprotein composed of an extracellular domain, N-and C-terminal anchors and two short intracellular domains [29]. Although other members of scavenger receptors class B, SRB-I and SRB-II, have been implicated in the uptake of *Mycobacterium fortuitum* into non-phagocytic cells [30], no role has been assigned to CD36 in innate immunity. We previously showed that CD36 is a selective co-receptor of TLR2 [16]. The role of CD36 as a co-factor for transmembrane proteins appears to be evolutionary conserved among species. Sensory neuron membrane protein (SNMP), a *Drosophila melanogaster* CD36 homolog, has a role in pheromone detection and acts as a co-factor for the OR67d/OR83 receptor [31]. However, the mechanisms underlying these interactions remain unclear.

In this study, we addressed the mechanism by which CD36 cooperates with TLR2. To investigate the role of CD36 as a TLR2 co-receptor, mCD36ED was cloned and expressed using a baculovirus expression system. Highly purified mCD36ED was obtained after three purification steps: immobilised metal affinity chromatography (IMAC), cationic exchange chromatography and size exclusion chromatography. Purified mCD36ED behaved as a monomer in gel filtration chromatography and its CD spectrum was consistent with folded protein with α-helical and β-sheet secondary structure. We also showed that recombinant mCD36ED contains N-linked glycans and disulfide bonds. We employed native PAGE, which has been successfully applied to investigate the binding of toll-like receptor 4/ myeloid differentiation factor 2 (TLR4/MD-2) with LPS (lipopolisaccharide) [32] and CD14 with LTA [33], to show that mCD36ED binds Pims, LTA, FSL1-fluorescein, but not Pam_3_CSK_4_. This selective binding displayed by mCD36ED explains the enhanced NF-κB activation by specific TLR2 ligands, but not others. Thus, the monomeric form of mCD36ED can bind TLR2 ligands in the absence of any other receptors or co-receptors. Furthermore, it was recently reported that monomeric transmembrane rat CD36 binds acetylated and oxidized low density lipoproteins [34].

The substantial increases in electrophoretic mobility shown by LTA-CD36 and Pim2-CD36, and several bands shown for the Pim4-CD36 complex, indicate that CD36 is able to bind more than one molecule of each of these ligands which was also confirmed by shifts in size exclusion chromatography for mCD36ED-Pim2 and mCD36ED-LTA complexes. These results are consistent with the role of CD36 as a scavenger receptor that sequesters glycolipid ligands. This selective binding displayed by mCD36ED explains the enhanced NF-κB activation by specific TLR2 ligands, but not others. Comparison of TLR2 ligands that were able to bind mCD36ED, versus those that were not, reveals that mCD36ED ligands have negatively charged moieties (polyglycerophosphates and *myo-*inositol phosphate for LTA and Pims, respectively), in agreement with reports that CD36 binds negatively charged ligands, such as phospholipids and phosphocholine [12]. These combined results are consistent with the role of mCD36ED as a scavenger receptor that sequesters polyanionic ligands [35]. Although CD36 binds Pim2 and Pim4, these synthetic phosphatidylinositol mannosides could not stimulate secretion of TNF-α by macrophages from C57BL/6 mice. Lipoarabinomannan (LAM) and its precursors have been reported to activate cells via the TLR2/TLR1 heterodimer [36]. Thus, this lack of activity for Pim2 and Pim4 may arise from the lack of the third acyl chain in these synthetic Pims that is necessary for interaction with TLR1 [37]. Although these synthetic Pims were not active, lipomannan from *Mycobacterium smegmatis* could induce TNF-α secretion in a TLR2- and CD36-dependent manner.

Because of the importance of TLR2 in the control of mycobacterial infections [38], [39], it will also be important to evaluate the role of CD36 in the pathogenesis of *Mycobacterium tuberculosis* and *Mycobacterium leprae*. In CD36 binding studies, we employed the same synthetic Pim2 that was loaded onto CD1d for determining the crystal structure of the Pim2-CD1d complex [40] and the same Pim4 structure that was reported to be a natural ligand of CD1d [41]. Furthermore, CD36 may also play a role in the endocytosis of Pims for presentation by CD1d, as with mannose receptor in the presentation of LAM by CD1b [42]. Pim2 shares the phosphatidylinositol diacylglycerol structure with the glycosylphosphatidylinositol (GPI) anchors from *Toxoplasma, Plasmodium, Leishmania and Trypanosome*, that could result in binding of GPI to mCD36ED. In fact, a recent report showed that secretion of TNF-α by GPI from *P. chabaudi chabaudi* AS is impaired in *Cd36−/−* macrophages, compared with wild-type macrophages. *Cd36−/−* mice also showed a higher level of parasitemia and mortality than wild-type mice [18]. Therefore, it is highly possible that CD36 cooperates with TLR2 in the recognition of GPI by binding and accumulating GPIs from parasites. It would be relevant to investigate if *Cd36−/−* mice infected with *Leishmania major, Toxoplasma gondii* and T*rypanosoma cruzi*, display higher levels of parasitemia and/or mortality, such as for *P. chabaudi chabaudi* AS.

Although the relevance of CD36 in the control of infection has been demonstrated, little is known about the mechanism by which CD36 enhances the response to TLR2 ligands. Our data reveal that response to lipomannan, LTA and FSL-1-fluorescein is dependent on the presence of TLR2, CD14, and CD36. In the case of LTA, loss of expression of functional CD36, CD14 or TLR6 caused a decrease in the ability of macrophages to activate the MyD88 signaling pathway. Furthermore, addition of either 50 or 100 ng/ml of mCD36ED increased the sensitivity of C57BL/6 macrophages to low concentrations of LTA , and restores the secretion of TNF-α by LTA from *oblivious macrophages*, but failed to restore the secretion of TNF-α from either *heedless* or *insouciant* macrophages. Increase in TNF-α secretion by LTA in the presence of mCD36ED is TLR2-dependent, as no increase in TNF-α secretion was observed in macrophages from *Tlr2* −/− mice. Recently, soluble CD36 in plasma was reported to be increased in type II diabetic patients and patients with polycystic ovarian syndrome [43], [44]. Because our data indicate enhancement of TNF-α secretion in C57BL/6 mice by mCD36ED at low concentrations of LTA, the presence of high levels of soluble CD36 in plasma from type II diabetic patients could contribute to exacerbation of the inflammatory response of macrophages in the presence of negatively charged diacylglycerol ligands and, therefore, may play a role in atherosclerosis development in diabetic patients.

Interestingly, our data indicate that mCD36ED is not able to restore TNF-α secretion in the absence of CD14. Thus, the main function of CD36 is to bind and transfer diacylglycerol ligands onto TLR2, in a CD14-dependent manner. This transfer of ligands onto CD14 by CD36 is also consistent with our immunoprecipitation results which show that mCD36ED does not directly interact with mTLR2ED/Fc.

Furthermore, CD14 also binds LTA, as well as LPS [34], [45]. The crystal structure of the murine CD14 ectodomain shows a possible hydrophobic pocket in its N-terminal region, which could be involved in ligand binding [46]. Soluble CD14 or transmembrane CD14 can bind monomers of LPS and transfer them to TLR4/MD2 complex [47]-[49]. In a similar way, CD14 could be a “shuttle” that takes monomers of diacylglycerol ligands, which are bound to CD36, and transfer them to TLR2/TLR6. Addition of mCD36ED did not restore TNF-α secretion by *insouciant* macrophages, indicating that CD36 cannot replace the function of TLR6 as a TLR2 co-receptor. In summary, we show that the TLR2 co-receptor role of CD36 is dependent only on the ectodomain of CD36, but not dependent on either signaling or endocytosis mediated by the intracellular domains of CD36. Therefore, our data are consistent with activation of the TLR2-dependent MyD88 signaling pathway by LTA from the plasma membrane [19], [20]. Similarly, activation of the MyD88 signaling pathway by LPS was also reported to be independent of LPS internalization [50], [51]. Based on our data, we propose a model in which CD36 binds diacylglycerol ligands, transfers them to CD14, which then loads these ligands onto TLR2/TLR6 (FSL-1, MALP-2, and LTA), or TLR2/TLR1 (lipomannan). Whether the transfer of pheromones from SNMP to OR67d is a direct or an indirect process remains to be shown.

## Materials and Methods

### Ligands, H5N1 Viet04 hemagglutinin and KRN TCR employed in native PAGE

Pam_2_CysSK_4_, Pam_3_CysSK_4,_ Pam_3_CysSK_4_-fluorescein, FSL-1 -fluorescein were obtained from EMC Microcollection (Tubingen, Germany). Purified LTA from *S. aureus*, lipomannan *and* FSL-1 was obtained from Invivogen (San Diego, CA, USA). Influenza hemagglutinin from the H5N1 Viet04 strain was a gift from Dr. James Stevens in our laboratory. The hemagglutinin precursor, corresponding to the H5N1 Viet04 strain, with a C- terminal His-tag was cloned in pAcGP67A (BD Biosciences, USA) for expression in insect cells. A thrombin site was included to release the C-terminal His-tag and a foldon was also included to help in the trimerization process. H5N1 Viet04 hemagglutinin was expressed using a baculovirus/ insect cell expression system and purified from culture supernatants, as previously described [27]. Synthetic phosphatidylinositol mannosides (Pim2 and Pim4) were synthesized as described previously [52]. cDNA for the α and β chain of KRN TCR were individually subcloned into the fly TCR expression vector pRMHa3. The final constructs code for the α1α2 and the β1β2 domains, respectively, followed by a linker sequence (SSADL), a thrombin site (LVPRGS), a leucine zipper (acidic for the α chain, basic for the β chain) and a hexa-histidine tag (C-terminal His-tag). Vectors were co-transfected into Drosophila Scheneider-2 insect cells along with a vector encoding a puromycin-resistance gene and stable cell-lines were established. Soluble TCRs were purified from culture supernatants, as previously described [53].

### Cell culture and cell lines

Sf-9 and Hi-5 insect cells were purchased from Invitrogen (USA) and employed for baculovirus generation and protein expression, respectively. Cell lines were cultured in suspension cultures in serum-free HyQ media (Hyclone, USA) at 27°C shaking at 225 rpm. Drosophila Scheneider-2 insect cells (Invitrogen, USA) were cultured in serum-free SFX insect express media (Hyclone, USA) in roller bottles. DMEM supplemented with 5% FCS and 2% penicillium streptomycin was used for culturing macrophages.

### Mice

C57BL/6, *insouciant* (TLR6*^int^*), *oblivious* (CD36*^obl^*), *heedless* (CD14*^hdl^*), *Tlr1* ^−/−^ and *Tlr2* ^−/−^ mice were maintained and bred in The Scripps Research Institute Vivarium under the supervision of the Department of Animal Resources. All studies involving mice were performed in accordance with the rules of Institutional Animal Care and Use Committee of The Scripps Research Institute. All experiments were performed according to the US National Institutes of Health guidelines.

### Cloning and expression of mCD36ED

The mCD36ED was amplified by PCR from a vector harboring the complete murine CD36 DNA (CMV CD36-pCDNA3.2/V5-DEST2) and the following primers:

Forward primer: GTG TGT **GGA TCC** CGG AGA CAT GCT TAT TGG GAA GAC AAT CAA AAG GG and Reverse primer: GTG TGT GAA **GCGGCC GC TCA** GTG ATG ATG ATG A TG ATG CTTG ATT TTC CCA GTC ACT TGT GTT TTG AAC. In the forward primer, a *Bam*HI restriction enzyme site was included (indicated in bold), while in the reverse primer a *Not*I (indicated in bold) restriction site and a stop codon (indicated by underline) was inserted. PCR amplification was performed using GC- rich DNA polymerase (Roche, USA) and the following steps were carried out: initial denaturation at 95°C for 4 minutes, denaturation for 35 cycles of 45 seconds at 95°C, annealing for 1 minute at 65°C, polymerization for 4 minutes at 72°C, final elongation for 7 minutes at 72°C.

The PCR product was purified by gel extraction, cloned into XL PCR Topo cloning vector (Invitrogen, USA) and subsequently subcloned into the *Bam*HI and *Not*I sites of the baculovirus transfer vector pAcGP67A (BD Biosciences, USA) for expression of mCD36ED with a C-terminal His-tag (Gen Bank accession number: GQ227601). The correct mCD36ED DNA sequences were confirmed at each step by sequencing both DNA strands. 2 µg of mCD36ED pAcGP67A plasmid DNA was co-transfected with 2 µg of Profold ER1 baculovirus DNA (AB vector, USA) in Sf-9 cells. After 5 rounds of viral amplification, a titer of 1×10^9^ virus/ml was obtained as determined by an end point dilution assay. Baculovirus infection was monitored by GFP expression, which was encoded in the Profold ER1 baculovirus DNA. For large scale mCD36ED protein expression, 3 liters of Hi-5 cells were infected with mCD36ED Profold ER1 baculovirus at an MOI of 3 and harvested after 7 days. The first purification was done by IMAC using Ni-NTA beads (Qiagen, USA) and a Western blot was performed to confirm the presence of mCD36ED in the elution from the Ni-NTA beads. After SDS PAGE, the proteins were transferred to a PVDF membrane by electro-blotting for 1 hour at 100 volts. The membrane was blocked with 1 % BSA, 0.1% Tween 20 for 1 hour at room temperature and incubated overnight at 4°C with an anti penta-His tag antibody (1/100 dilution; Calbiochem, USA). A second incubation with an anti-mouse IgG-peroxidase conjugated antibody (1/5000 dilution; Pierce, USA) was performed for 1 hour at room temperature. After developing the Western blot with ECL (Pierce, USA), a band between 50 and 75 kDa was observed.

### CD spectroscopy of mCD36ED

mCD36ED at a concentration of 0.14 mg/ml in 50 mM NaCl, 10 mM TrisCl pH 8 was used for circular dichroism (CD) experiments. CD spectra of mCD36ED were recorded at 25°C using an AVIV 202 spectropolarimeter (Hellma, Mullheim, Baden, Germany). The scans were performed in triplicate from 260 to 195 nm with 1 nm resolution. The spectra from triplicate scans were averaged and the final spectra were obtained by subtracting the buffer measurement spectra obtained under identical conditions. Results are expressed as molar ellipticity per residue. Protein concentrations were determined using the Bradford method (Bradford solution, Pierce, USA). Typical CD spectra of proteins rich in α-helical (Che a 3, [54]), β-sheet (human TLR-2 ectodomain, [55]), and mixed α/β secondary structure (Bet v 1, [56]) are shown as reference.

### Determination of N-linked glycosylation and disulfide bonds in mCD36ED

To investigate the presence of disulfide bonds in mCD36ED, 20 µg of mCD36ED was incubated at 100°C in SDS buffer with or without β-mercaptoethanol for 5 minutes and the samples were subjected to SDS PAGE for 1 hour at 200 volts. To demonstrate the presence of N-linked glycosylation, 10 µg of mCD36ED was deglycosylated with 50 units of PNGase F under native conditions. The difference in electrophoretic mobility was indicative of the presence of N-linked glycans in mCD36ED.

### Native PAGE and gel filtration experiments

To determine binding of mCD36ED to different ligands, 10 µg of mCD36ED was incubated overnight at 37°C with 1 µg of synthetic Pim4, 1 µg Pim2, 1 µg LTA, 1 µg of Pam_3_CSK_4_ fluorescein, 10 µg of Viet 04 hemagglutinin, or 1 µg FSL-1 fluorescein. The molar ratio of trimeric hemagglutinin to mCD36ED is approximately 0.3 and the molar ratio of the monomeric HA to mCD36ED is approximately 1. In the case of the lipid ligands, it was added an excess of ligands respect to mCD36ED. The molar ratios ligand/mCD36ED were: 3.9 (Pam_3_CysSK_4_), 5.6 (FSL-1 fluorescein), 3.2 (Pim2), 3.9 (Pim4), 2.5 (LTA). For negative control, 10 µg of KRN TCR was incubated for one hour at 37°C with 1 µg of synthetic Pim4, 1 µg Pim2, 1 µg LTA, 1 µg of Pam_3_CSK_4_, or 1 µg FSL-1. The molar ratios of ligand/ KRN were: 4.0 (Pam_3_CysSK_4_), 5.8 (FSL-1), 3.3 (Pim2), 4.0 (Pim4), 2.6 (LTA). The samples were run for 4 hours at 100 volts in a native 4–20% polyacrylamide gel (Criterion Tris HCl gel, Biorad) at room temperature with a Glycine Tris running buffer at pH 7.4. Fluorescence was determined using a Versadoc imaging system and the gel stained with Coomassie Blue. To determine if binding altered the retention volume of mCD36ED, 100 µg of mCD36ED were incubated overnight at 37°C with 1 µg of LTA, Pam_3_CSK_4_ or Pam_2_CSK_4_. The samples were loaded on a Superdex 200 10/30 column (Pharmacia). The gel filtration chromatography was performed at room temperature using PBS pH 7.4 as running buffer at a flow rate of 0.5 ml/min and a fractionation volume of 0.5 ml

### Dot blots experiment to detect LTA in mCD36ED-LTA complex purified by size exclusion chromatography

The collected fractions of CD36-LTA and CD36 from Superdex 200 10/30 were used for dot blots with an anti LTA antibody (clone 55, Hycult, Netherland) that detects the LTA polyglycerophosphate moiety. The first step was to blot 3 µL CD36 LTA, 3 µL of CD36 in a PDVF membrane, previously activated with 100% methanol. The membrane was blocked with 1 % BSA, 0.1% Tween 20 for 1 hour at room temperature and incubated overnight at 4°C with a 1/1000 dilution of an anti-LTA antibody (1/1000 dilution; clone 55, Hycult, Netherland). A second incubation with a goat anti-mouse, IgG -peroxidase conjugated antibody (1/5000 dilution; Pierce, USA) was performed for 1 hour at room temperature. The dot blot was developed with ECL (Pierce, USA). The anti-LTA antibody and LTA were employed as positive controls, while Pam_2_CSK_4_ and PBS were employed as negative controls in the dot blot experiment.

### Determination of secretion of TNF-α by macrophages

Macrophages from the different mutant mice were induced by addition of 3% thyoglycollate. After 3 days, peritoneal macrophages were harvested and 50,000 macrophages per well (macrophages harvested from the same mouse were seeded in duplicate wells) were incubated in DMEM with 5 % FCS and 2 % penicillium streptomycin at 37°C, after which culture media were discarded and replaced with 100 microliters of fresh media and incubated for 4 hours with different concentrations of bacterial ligands or different concentrations of LTA plus a constant amount of active mCD36ED (50 or 100 ng/ml), or heated denatured mCD36ED (100 ng/ml). Subsequently, the supernatants were harvested and the values of TNF-α were determined by an L929 cytotoxic assay. An average value of TNF-α was obtained for each duplicate wells (TNF-α duplicate wells) and the reported TNF-α values were the average values of the TNF-α duplicate wells. Because the data of each duplicate wells represent the secretion of TNF-α from macrophages of a particular mouse, the duplicate wells are independent of each other. Therefore, the number of independent replicates per experiment is the number of mice per experiment. In order to determine the effect of the different mutants in the response to lipomannan, FSL-1-fluorescein and LTA, and for determination of the activity of mCD36ED, macrophages were extracted from two and three mice, respectively. Values are expressed as mean values +/− SEM. All experiments were repeated three times. Therefore, the data are representative from three independent experiments.

### Immunoprecipitation

To determine any direct interaction between the ectodomains of murine CD36 and TLR2, 24 µg of mCD36ED was incubated with 10 µg of mTLR2ED/FC chimera overnight at room temperature in PBS pH 7.4, and then incubated with 50 µl of magnetic protein G beads (NEB, USA) for 1 hour at 4°C. The supernatant was removed and the protein beads were washed 5 times with PBS. The proteins were eluted from the beads by heating for 5 minute at 100°C with reducing loading buffer and run on an SDS PAGE.

## Supporting Information

Figure S1 Characterization of recombinantly expressed mCD36ED. (A) Western blot showing the presence of mCD36ED after Ni-NTA purification of supernatants of Hi-5 cells infected with mCD36ED ER1 Profold baculovirus. Lanes: 1- Molecular weight markers; 2- Supernatant of non-infected Hi-5 cells; 3- Elution from Ni-NTA beads of supernatant from Hi-5 cells infected with mCD36ED ER1 Profold baculovirus. (B) The identity of mCD36ED was confirmed by mass spectrometry. To confirm that the purified protein was indeed mCD36ED, the gel bands from figure 1B were excised, reduced with DTT (10 mM), digested with trypsin overnight before being analyzed by nano LC-MS/MS (TSRI Center for Mass Spectrometry). The 6 peptides, which were identified using MASCOT, are depicted in red.
(1.03 MB TIF)

Figure S2 mCD36ED has intramolecular disulfide bonds and N-linked glycosylation. (A) SDS PAGE of mCD36ED under reducing and non-reducing conditions. Lanes: 1- mCD36ED under reducing conditions; 2- mCD36ED under non-reducing conditions. (B) 10 µg of purified mCD36ED (native and denatured) was digested with 50 units of PNGase F overnight at 37°C. The samples were reduced and run on an SDS PAGE.
(1.41 MB TIF)
