# Supplementary figures and images for: Soluble CD36 Ectodomain Binds Negatively Charged Diacylglycerol Ligands and Acts as a Co-Receptor for TLR2
Source: PLoS One. 2009 Oct 22;4(10):e7411. doi: 10.1371/journal.pone.0007411 (PMC2760212; doi:10.1371/journal.pone.0007411)

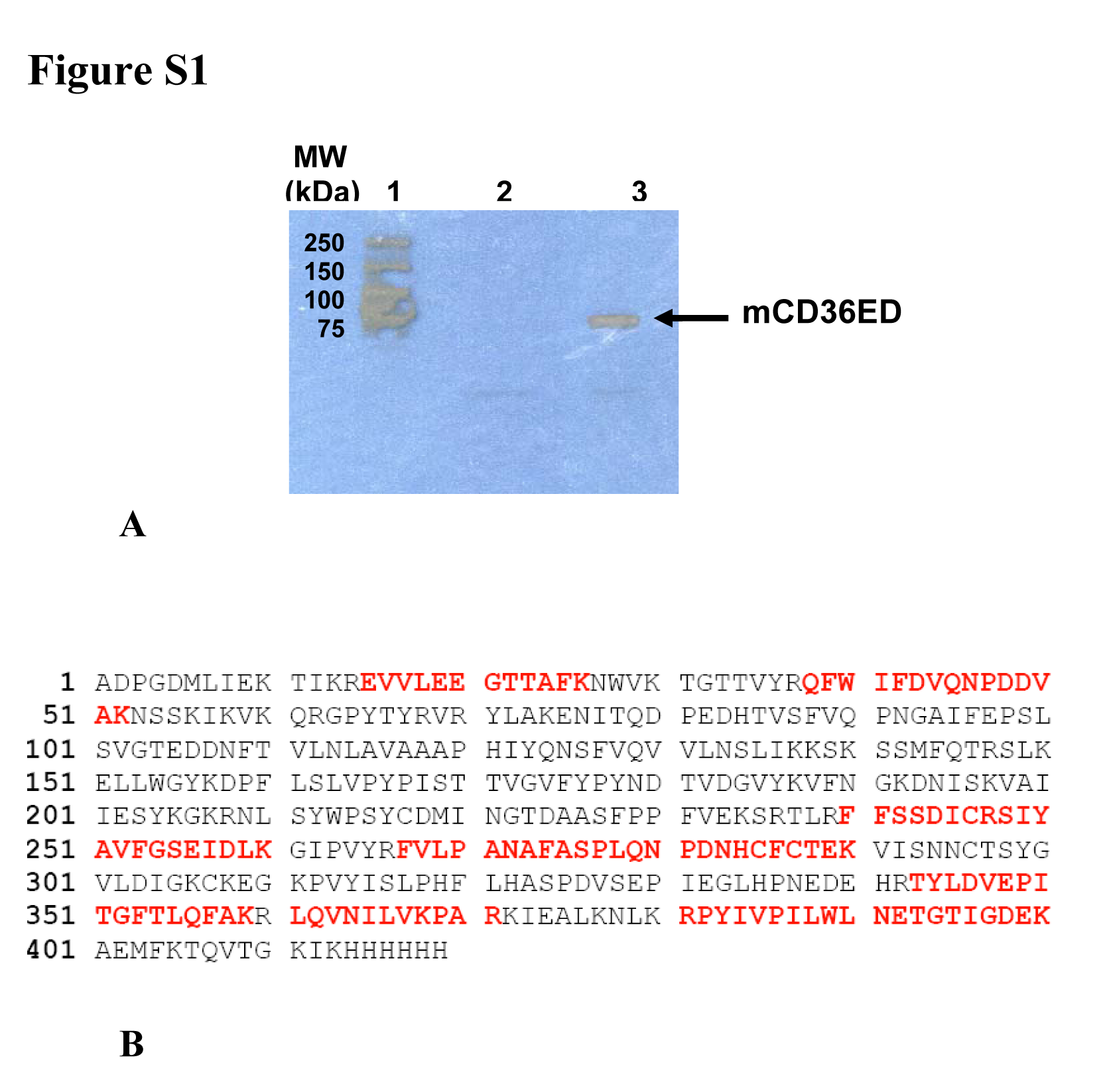

Supplement: Figure S1 — Characterization of recombinantly expressed mCD36ED. (A) Western blot showing the presence of mCD36ED after Ni-NTA purification of supernatants of Hi-5 cells infected with mCD36ED ER1 Profold baculovirus. Lanes: 1- Molecular weight markers; 2- Supernatant of non-infected Hi-5 cells; 3- Elution from Ni-NTA beads of supernatant from Hi-5 cells infected with mCD36ED ER1 Profold baculovirus. (B) The identity of mCD36ED was confirmed by mass spectrometry. To confirm that the purified protein was indeed mCD36ED, the gel bands from figure 1B were excised, reduced with DTT (10 mM), digested with trypsin overnight before being analyzed by nano LC-MS/MS (TSRI Center for Mass Spectrometry). The 6 peptides, which were identified using MASCOT, are depicted in red. (1.03 MB TIF) [file pone.0007411.s001.tif]

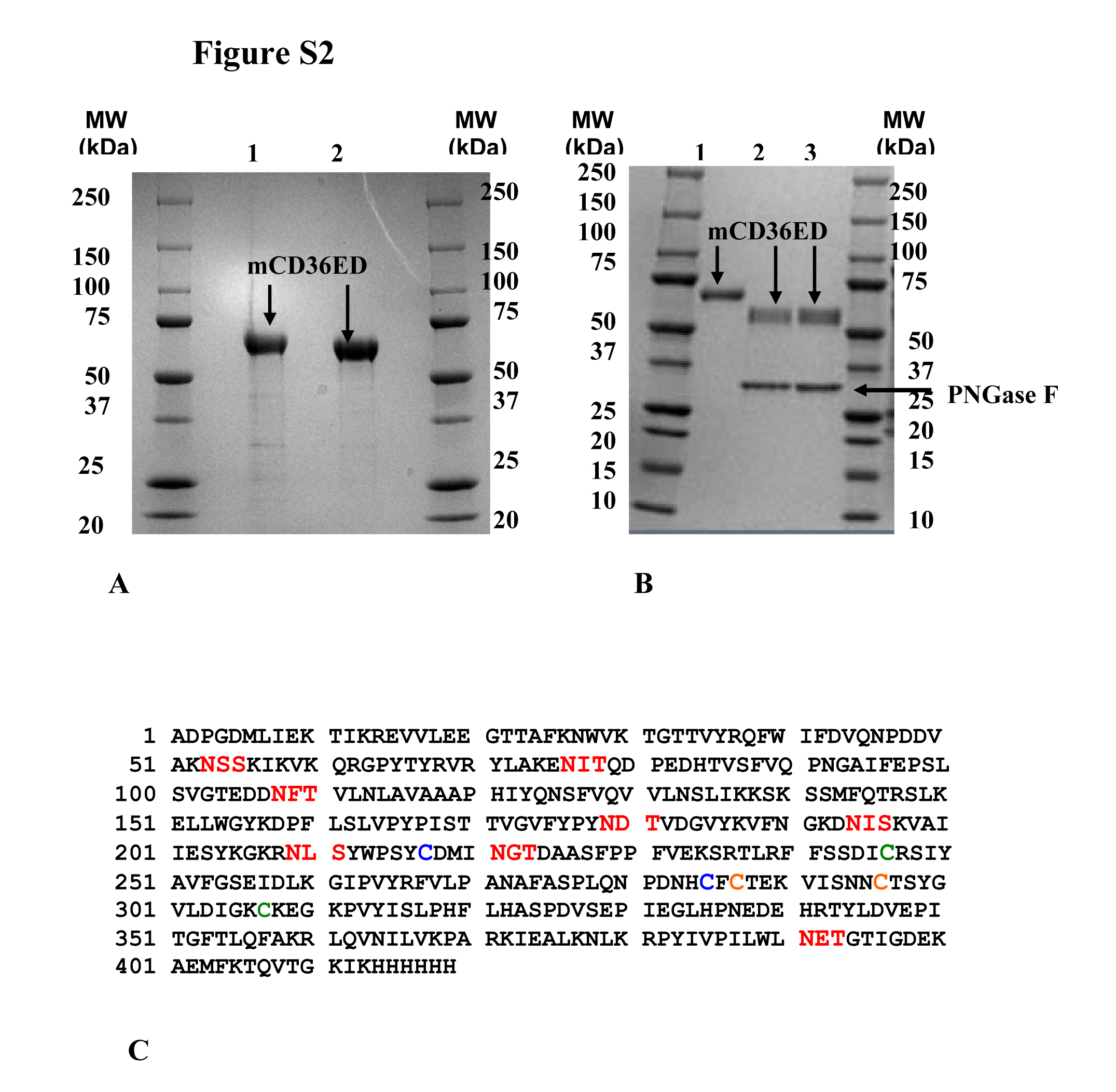

Supplement: Figure S2 — mCD36ED has intramolecular disulfide bonds and N-linked glycosylation. (A) SDS PAGE of mCD36ED under reducing and non-reducing conditions. Lanes: 1- mCD36ED under reducing conditions; 2- mCD36ED under non-reducing conditions. (B) 10 µg of purified mCD36ED (native and denatured) was digested with 50 units of PNGase F overnight at 37°C. The samples were reduced and run on an SDS PAGE. (1.41 MB TIF) [file pone.0007411.s002.tif]
